# The positioning of the asymmetric septum during sporulation in *Bacillus subtilis*

**DOI:** 10.1371/journal.pone.0201979

**Published:** 2018-08-09

**Authors:** Imrich Barák, Katarína Muchová

**Affiliations:** Institute of Molecular Biology, Slovak Academy of Sciences, Bratislava, Slovakia; Centre National de la Recherche Scientifique, Aix-Marseille Université, FRANCE

## Abstract

Probably one of the most controversial questions about the cell division of *Bacillus subtilis*, a rod-shaped bacterium, concerns the mechanism that ensures correct division septum placement–at mid-cell during vegetative growth but closer to one end during sporulation. In general, bacteria multiply by binary fission, in which the division septum forms almost exactly at the cell centre. How the division machinery achieves such accuracy is a question of continuing interest. We understand in some detail how this is achieved during vegetative growth in *Escherichia coli* and *B*. *subtilis*, where two main negative regulators, nucleoid occlusion and the Min system, help to determine the division site, but we still do not know exactly how the asymmetric septation site is determined during sporulation in *B*. *subtilis*. Clearly, the inhibitory effects of the nucleoid occlusion and Min system on polar division have to be overcome. We evaluated the positioning of the asymmetric septum and its accuracy by statistical analysis of the site of septation. We also clarified the role of SpoIIE, RefZ and MinCD on the accuracy of this process. We determined that the sporulation septum forms approximately ^1^/_6_ of a cell length from one of the cell poles with high precision and that SpoIIE, RefZ and MinCD have a crucial role in precisely localizing the sporulation septum. Our results strongly support the idea that asymmetric septum formation is a very precise and highly controlled process regulated by a still unknown mechanism.

## Introduction

The cell division processes in *E*. *coli* and *B*. *subtilis* have been studied intensively for decades. The earliest visible event in cell division is the formation of a Z-ring by FtsZ, a tubulin like protein, at the future septum site. The Z-ring appears to be an accurate marker for the position of the division site and is recognized by a set of cell division proteins–the divisome. At least two distinct mechanisms contribute to the precise placement of the division machinery: nucleoid occlusion and the Min system [1]. In *B*. *subtilis*, the main player of nucleoid occlusion is Noc; a DNA associated protein that blocks division from taking place over the chromosome [2]. The second mechanism, Min system, includes four proteins: MinC, MinD, DivIVA, and MinJ [3]. MinC is the actual cell division inhibitor that directly binds to FtsZ and is activated by MinD, a membrane-associated ATPase [4]. The localization cue for the MinCD complex is provided by MinJ, a MinD protein partner, which is recruited by DivIVA to the site of septation and to the poles [5,6]. This creates a relatively static MinC gradient to prevent additional septation in close proximity to the newly formed septum, as well as inappropriate minicell division at the cell poles [7]. MinD also has ATPase activity even though it does not drive a rapid oscillation of the protein from pole to pole as it is characteristic for *E*.*coli* MinD [8]. On the other hand, fast membrane dissociation and re-association of *B*. *subtilis* MinD was observed [9]. The biological role of this phenomenon is not clear, but the dynamics of MinD localization and its reversible membrane binding are integral to the function of both Min systems. The mid-cell division site is located with high precision; in both *E*. *coli* and *B*. *subtilis*, the standard deviation from the mid-cell position as measured from the cell pole was 0.01 (denoted as 0.50±0.01) [10,11]. Although both nucleoid occlusion and the Min system were proposed to play an important role in determining the precise septation site in *B*. *subtilis*, it was shown that deletion of both systems together does not greatly decrease this precision [12]. This suggests that other factors or mechanisms are required to allow the precise location of the septation site during vegetative growth. Among these, we must consider early cell division protein EzrA which seems to act as negative regulator of FtsZ assembly since cells lacking EzrA form multiple Z-ring not only at the mid-cell but also at polar sites [13]. It was also shown that these cells are longer than wild type cells because a delay in septum constriction [14]. In addition, the function of EzrA seems to be even more complex and EzrA is also involved in the recruitment of the main peptidoglycan synthesizing enzyme (PBP1) to the division site [15]. During its life cycle, wild-type *B*. *subtilis* can form both a mid-cell vegetative septum and also an asymmetric septum during a differentiation process called sporulation. Before this can happen, however, the Min system function that ensures that the Z-ring appears in the centre of the cell must be overridden. While the complete mechanism of this process is still unknown, partial answers have been provided by the observation that during this stage of development, the function of DivIVA switches from regulating cell division to allowing proper chromosome segregation to occur in the forespore, the smaller part of the cell arising after asymmetric cell division. This is likely accomplished by DivIVA switching its binding partner from MinJ to the DNA-binding RacA protein [16]. It is not known what the MinJ, MinC and MinD proteins are doing during this stage of sporulation. It was recently shown that MinJ and MinD could serve as part of an additional chromosome anchoring mechanism [17]. Although depletion of any of these proteins has no detectable effect on sporulation frequency [18], it is still not possible to exclude the possibility that the Min system has at least a partial role in sporulation. It was observed that in *minCD* mutant cells, a sporulation-like septum appears, but is misplaced from its normal polar site: in some cells it forms either at or near the centre of the cell [19,20].

The first clear morphological feature of sporulation in *B*. *subtilis* is the polar cell division that starts with migration of the Z-ring from mid-cell to the two cell poles on a spiral trajectory, in a process that depends on the sporulation-specific overexpression of *ftsAZ* and the presence of SpoIIE [21]. SpoIIE co-localizes with the polar Z-rings. One of the Z-rings matures into the sporulation septum while the other dissolves. Asymmetric cell division otherwise appears to involve the same set of divisome proteins used during vegetative cell division, though the resulting sporulation septum is much thinner. Interestingly, SpoIIE is the only sporulation-specific protein whose deletion or mutation causes substantial changes in the ultrastructure of the asymmetric septum. *spoIIE* null mutants are defective in sporulation, while expression at lower frequency gives rise to aberrantly thick asymmetric septa [22]. Furthermore, the absence of two SpoIIE partners, RodZ and DivIVA, causes considerable disturbance of asymmetric septum formation [20,23]. Additional sporulation-induced protein RefZ (Regulator of FtsZ) was discovered recently to facilitate the switch from a medial to a polar FtsZ ring placement at the onset of sporulation [24, 25]. RefZ is a DNA binding protein that binds to its cognate binding motifs (RBMs), localized near the asymmetric septation site, and promotes precise chromosome arm positioning during sporulation [25]. This mechanism was suggested to be one way the position of the sporulation septum is regulated [25]. It was shown that in the absence of RefZ, asymmetric septa formation two hours after sporulation initiation is only 75% of the wild type level [25].

The precise site where the sporulation septum forms has not yet been determined, nor has the accuracy of its positioning been determined, though it does seem to be formed with high accuracy near the cell poles. In this work, we show that the accuracy of the asymmetric septation positioning is comparable with that of the mid-cell septation process. We also show that SpoIIE, RefZ and MinCD have an important role in finding the site of asymmetric septum formation during sporulation. Nevertheless, we can still only speculate how it is that the cell finds the asymmetric division site with such high precision.

## Materials and methods

### Media and bacterial strains

*B*. *subtilis* cells were grown in Difco sporulation medium (DSM) supplemented with spectinomycin (100 μg ml^-1^), chloramphenicol (5 μg ml^-1^), kanamycin (10 μg ml^-1^, tetracycline (10 μg ml^-1^) or erythromycin (1 μg ml^-1^) and lincomycin (25 μg ml^-1^) when required [26]. P_xyl_-driven expression was induced using 0.1–0.5% xylose.

Strain IB1538 (*p*_*spoIIE*_*-spoIIEypet cat lacA*::*pxyl-cfp-rodZ erm*), in which SpoIIE fused to Ypet is produced under the control of its native promoter, and CFP-RodZ is produced under the control of a xylose-inducible promoter at the ectopic *lacA* locus, was prepared as described previously [23]. Strain PY180 (*spoIIE*::Tn*917*Ω*HU7*) was described previously [22]. Strain IB1723 (*p*_*spoIIE*_*-spoIIEypet cat refZ*::*tet*) was prepared by transformation of strain IB1537 (*p*_*spoIIE*_*-spoIIEypet cat*) [23] with BJH247 chromosomal DNA [24]. Strain IB1724 (*p*_*spoIIE*_*-spoIIEypet cat minCD*::*kan*) was prepared similarly by transforming IB1537 with IB1371 chromosomal DNA [27]. Strain IB1725 (*p*_*spoIIE*_*-spoIIEypet cat ezrA*::*tet*) was prepared by transformation of strain IB1537 (*p*_*spoIIE*_*-spoIIEypet cat*) [23] with chromosomal DNA from strain 3362 [15].

### Fluorescence microscopy and image acquisition

*B*. *subtilis* cultures were grown as liquid cultures as described above. Cells were inspected two hours after sporulation initiation. For membrane visualization, the fluorescent dye FM 4–64 (Molecular Probes) was used at concentrations of 0.2–1 μg ml^-1^. To visualize DNA cells were stained with 0.2 μg ml^-1^ DAPI. Cells were examined under the microscope on 1% agarose covered slides. When it was necessary to increase the cell density, cells were concentrated by centrifugation (3 min at 2,300 × g) and resuspended in a small volume of supernatant prior to examination. All images were obtained with an Olympus BX63 microscope equipped with a Hamamatsu Orca-R^2^ camera. Olympus CellP imaging software or Olympus Image-Pro Plus 6.0 software were used for image acquisition and analysis.

### Measurements of cell length, the position of SpoIIE, RodZ, and FM4-64, and calculation of statistics

Olympus CellP software was used to calculate cell lengths and measure the fluorescence signals from digital images. To highlight the edges of the measured cells, we used a Sobel filter, a non-linear method for highlighting edges comprised of a set of derivative filters. The Sobel filter generally yields a magnitude of difference and the direction of the most significant change. The filter uses two matrices to calculate values for *a* and *b* parameters. The square root of the sum of the squares of these parameters gives the intensity: √ (*a*^2^+*b*^2^).

The numerical values for each cell length and fluorescence signal were exported from the CellP software to Microsoft Excel, and the mean, standard deviation, standard error of the mean (SEM) and the number of cells evaluated were all calculated.

We scored the localizations of SpoIIE, RodZ and FM4-64 when the fluorescence signals were oriented perpendicularly to the long axis of the cell and the pixel position with highest signal intensity was taken as the site of septation. The localizations of signals arising from cells after stage IIi, where the signals begin to curve alongside the engulfing forespore membrane, were not scored and were not included in the localization analysis. The signal positions were expressed as a fraction of the cell length by measuring the position of the maximum signal to the closest cell pole, then dividing by the total cell length. Statistical analyses were carried out using Microsoft Excel and R [28]. Statistical analyses included Student’s t-test and the Kolmogorov-Smirnov test. All statistics was performed using a 95% confidence interval, where p-value <0.05 indicates a statistically significant difference between the compared groups. All calculated p-values were below 1.10^−12^.

## Results

### Sporulation septum positioning

**Fig 1:**
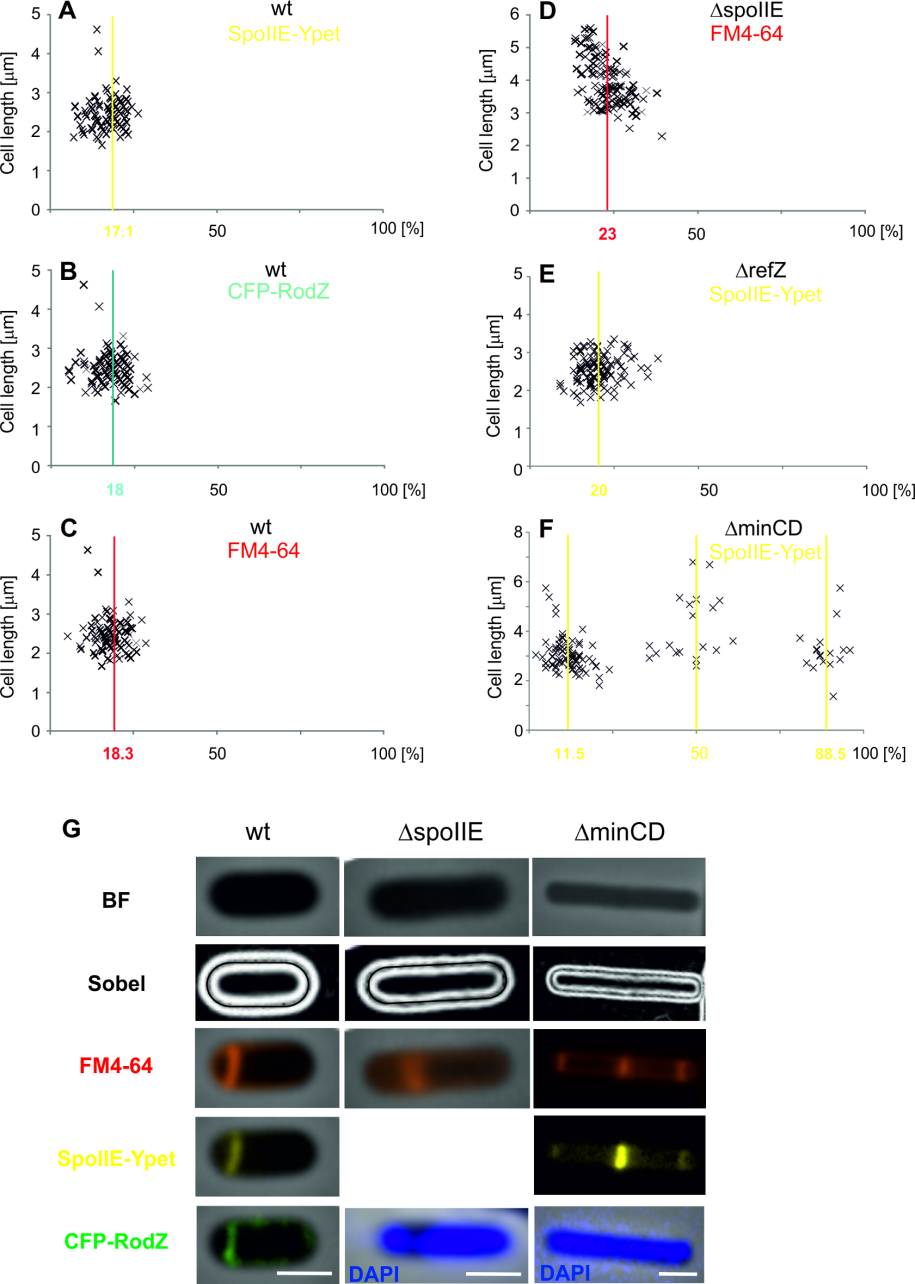
The sporulation septum position in wild type, ΔSpoIIE, ΔRefZ and ΔMinCD strains. The average position of the sporulation septum is measured from the nearest cell pole, and expressed as a percentage of the total cell length (x-axis). Y-axis represents the cell length in μm. (A) The sporulation septum position in IB1538 based on the SpoIIE-Ypet signal. (B) The sporulation septum position in IB1538 based on the CFP-RodZ signal. (C) The sporulation septum position in IB1538 based on the FM4-64 membrane dye signal. (D) The sporulation septum position in the PY180 (*ΔspoIIE*) strain based on the FM4-64 membrane dye signal. (E) The sporulation septum position in IB1723 (*ΔrefZ*) based on the SpoIIE-Ypet signal. (F) The sporulation septum position in IB1724 (*ΔminCD*) based on the SpoIIE-Ypet signal. (G) Example images showing cell length using a Sobel filter and sporulation septum position signals from SpoIIE-Ypet, CFP-RodZ and FM4-64 in wild type, PY180 (*ΔspoIIE*) and IB1724 (*ΔminCD*) strains as described in Materials and Methods. In addition, there are DAPI staining of chromosomal DNA in PY180 (*ΔspoIIE*) and IB1724 (*ΔminCD*) strains to show that asymmetric septation started after initiation of sporulation when the nucleoid forms an axial filament from pole to pole. The scale bar represents 1 μm.

Formation of an asymmetric septum is the first clear morphological sign of sporulation in *B*. *subtilis*. The multifunctional protein SpoIIE is a crucial protein for the formation of this sporulation septum. The earliest event of asymmetric division is movement of FtsZ towards two polar sites on a helical trajectory [21]. Here, the FtsZ oligomers are stabilized as a Z-ring by SpoIIE [29]. SpoIIE localization therefore serves as a good marker for determining the septation site, so we used the localization of a SpoIIE-Ypet fusion to determine the position of the sporulation division site. SpoIIE forms an E-ring at the same site as the Z-ring during sporulation stage I [30]. SpoIIE remains at the septation site even after the septum has formed (stage IIi), unlike the Z-ring, which dissociates from this site and dissembles. We examined SpoIIE localization in *B*. *subtilis* strain IB1538, which holds a SpoIIE fused to Ypet, a photostable monomeric derivative of YFP [31]. In this strain, the *spoIIE-ypet* fusion is used to replace the cell’s original copy of the *spoIIE* gene, thereby keeping the fusion under the control of the native *spoIIE* promoter. This fusion protein seems to be functional, and IB1538 sporulates with comparable efficiency as the wild type PY79 strain (sporulation 87% of the wild type). A SpoIIE-Ypet fusion is more suitable for evaluating the localization of the asymmetric septation site than an FtsZ-YFP fusion, because the latter, by itself, does not allow cell division [13]. It was previously shown that SpoIIE interacts and co-localizes during the early stages of sporulation with RodZ [23]. We therefore used a CFP-RodZ fusion as a second marker, also in IB1538. One additional marker, the membrane-binding FM 4–64 dye, was also used to locate the sporulation septum. Cells were inspected two hours after sporulation initiation, and SpoIIE-Ypet and CFP-RodZ were detected in more than 60% and 98% of cells, respectively. Only those cells in stage I or IIi of sporulation (>10% of at least 1000 cells), as defined by Illing and Errington [30], were used to determine the asymmetric septum position. In these stages, both the SpoIIE-Ypet and CFP-RodZ fluorescence signals form clear and straight lines when viewed under a microscope (Fig 1); cells in stage IIii and later begin to show curved signals, making exact localization difficult. The average SpoIIE-Ypet position, measured from the nearest cell pole and expressed as a fraction of the total cell length, was 0.171 ± 0.04 (mean SpoIIE position ± standard deviation (SD); 112 cells; Fig 1A). In 74% of cells, SpoIIE localized within ± 5% a cell length of the average position. This shows that the cell positions the asymmetric septum to the same relative position within the cell with high precision during sporulation. On the other hand, this is a lower precision than that reached by wild-type *B*. *subtilis* cells when placing the Z-ring at the mid-cell site. In that case, 91% of cells put the Z-ring within ± 5% of the mid-cell (0.5 ± 0.05) [11]. Similar results were obtained from the CFP-RodZ and FM 4–64 signals: CFP-RodZ appeared at 0.18 ± 0.05 (mean RodZ position ± SD; 109 cells), while FM 4–64 appeared at 0.183 ± 0.04 (107 cells; Fig 1B and 1C).

The average length of the evaluated cells was 2.46 ± 0.4 μm and the average septum location is 0.45 ± 0.12 μm from the nearest pole (Table 1).

**Table 1:** Summary of sporulation septa position in wild type and mutant strains.

Strain	Cell length	Distance from	Relative distance from
	± SD [μm]	the pole ± SD [μm]	the pole ± SD [%]
wt	2.46 ± 0.4	0.42 ± 0.13	17.1 ± 4.3
ΔspoIIE	3.94 ± 0.7	0.89 ± 0.17	23.2 ± 5.3
ΔrefZ	2.54 ± 0.4	0.52 ± 0.17	20.0 ± 5.3
ΔminCD	3.05 ± 0.7	0.34 ± 0.12	11.5 ± 4.2

SpoIIE-Ypet position was used in all cases except Δ*spoIIE* in which FM 4–64 signal served for septum localization. All numbers represent averages from all cells counted with standard deviation (SD). Distances are from the nearest cell poles.

**Fig 2:**
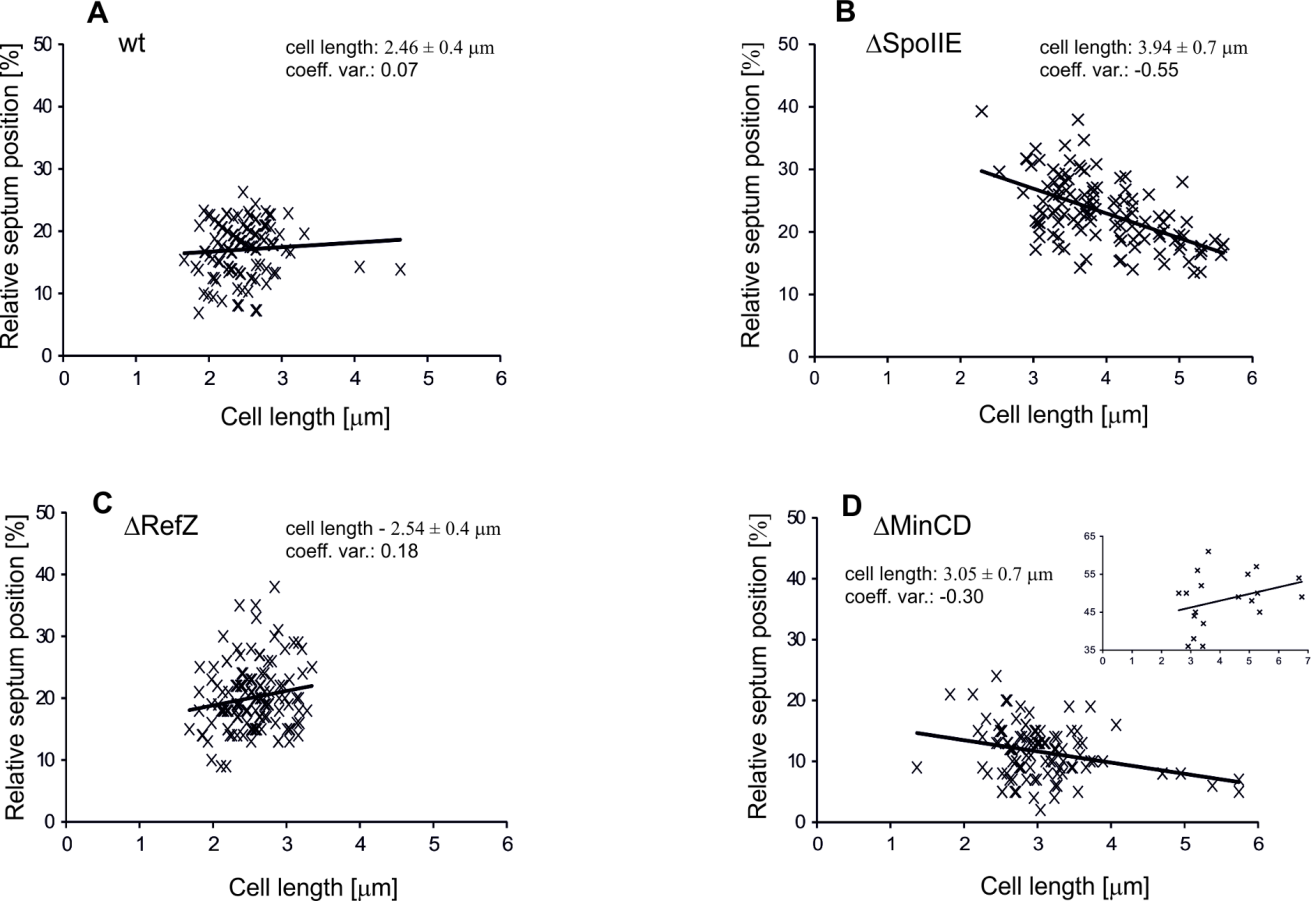
Scatter plots. Plots showing the asymmetric septum location in fractions of cell length in the wild type, ΔSpoIIE, ΔRefZ and ΔMinCD strains.

By expressing all three of these results as a fraction, we may say that the sporulation septum tends to appear, with relatively high precision, approximately ^1^/_6_ (strictly, ^1^/_5.45_−^1^/_5.85_) of the total cell length from one of the cell poles. In all three cases, the exact position seems to be independent of cell length: the highest linear correlation coefficient was 0.07 (Fig 2A).

### Asymmetric septum positioning in the absence of SpoIIE

It was previously shown that *spoIIE* null mutant strains have a noticeably reduced frequency of asymmetric septum formation (50–70% are aseptate cells), indicating that SpoIIE is required for efficiently initiating septum formation. In addition, TEM images show that the ultrastructure of these septa are also different, more closely resembling vegetative septa, which are thicker than the sporulation septa. Finally, these mutant strains cannot form spores [22,30]. While SpoIIE is clearly important for forming sporulation septa properly and efficiently, it had not previously been shown where asymmetric septa localize in cells lacking it. For this reason, we measured the localization of septa in PY180, a Δ*spoIIE* strain [22]. Because the GFP-RodZ signal becomes dispersed throughout the cell when SpoIIE is missing, and no fluorescence signal from it can be observed at sporulation septa [23], we used the membrane-binding FM 4–64 dye to identify the sites of asymmetric septation. We found that the septa appear at 0.23 ± 0.05 cell lengths (mean FM 4–64 position ± SD; 119 cells) from the nearest cell pole, which is slightly less than ^1^/_4_ (specifically ^1^/_4.3_) of the cell length (Fig 1D). In this deletion strain, only 46% of cells positioned the septa within ± 5% of the average position of septa as in wild type strain, indicating that the cell positions the asymmetric septation site with lower precision when SpoIIE is absent. The cells of PY180 strain are also notably longer than wild type cells, with an average cell length of 3.9 ± 0.7 μm compared to 2.46 ± 0.4 μm for the wild type. The average septum location is 0.89 ± 0.17 μm from the pole compared to 0.45 ± 0.12 μm for the wild type (Table 1). In this case, the position of the asymmetric septum does appear to be moderately correlated with cell length: the linear correlation coefficient between position and cell length is -0.55 (Fig 2B), indicating that longer cells tend to position their septa relatively closer to the cell pole. From this, it appears that SpoIIE is necessary for both proper septum formation and precisely positioning the septum. *ΔspoIIE* cells tend to position the asymmetric septum farther from the cell pole than the wild type cells (0.23 versus 0.18 cell lengths). The septum is also placed less precisely than in wild type cells: only 64% of *ΔspoII*E cells have their asymmetric septum within ± 5% cell lengths of the mean septum position, in contrast to the 74% of wild type cells. Moreover, wild type cells show no linear correlation between their length and the location of their asymmetric septa, with the highest linear correlation coefficient being 0.07, while *ΔspoIIE* cells do show a moderate, negative linear correlation (CC = -0.55); that is, longer cells tend to have septa in a relatively shorter distance from the cell pole. Over 100 cells were scored in both groups.

### Asymmetric septum positioning in the absence of RefZ

**Fig 3:**
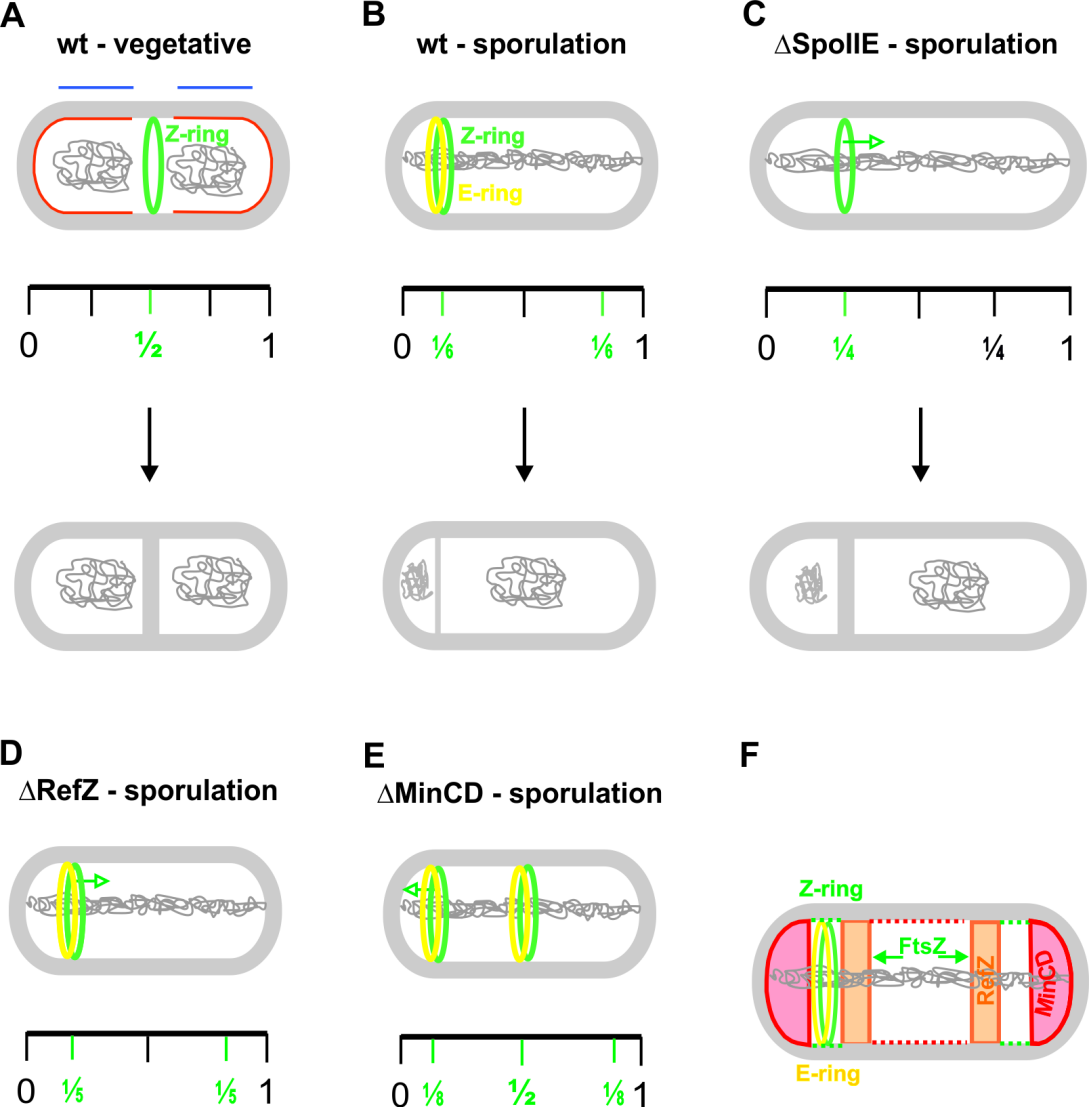
Models of division site positioning during vegetative growth and sporulation in *B*. *subtilis*. (A) During vegetative growth, upon initiation of DNA replication, the future division site is marked by a putative, but not yet identified, mid-cell defining factor. Upon segregation of the chromosomes (shown in grey inside of the cells), the nucleoid occlusion factors (blue lines above the nucleoids) clear the mid-cell site, while the Min system (shown in red) blocks Z-ring formation at the cell poles. The septum is formed with high precision at the mid-cell site. (B) During sporulation, the Z-ring forms approximately ^1^/_6_ of a cell length from one of the cell poles with high precision. In this cell cycle stage, the replicated chromosomes are in the form of axial filaments, and the Min system proteins are localized at the cell poles. It is not known how Z-ring formation prevails over the negative regulation of nucleoid occlusion and the Min system. (C) SpoIIE has a crucial role in precisely localizing the sporulation septum; in its absence, the asymmetric septum forms in an average of ^1^/_4_ a cell length from the nearest cell pole. This septum is thicker than the wild type sporulation septum and resembles the vegetative like septum. ΔSpoIIE cells cannot form spores. (D) RefZ has a moderate influence on the localization of the sporulation septum; in its absence the septation position moves farther from the cell pole, to an average position of ^1^/_5_ of the cell length. (E) MinCD influences the localization of the sporulation septum, and in cells lacking these proteins the septum position moves much closer to the cell pole, to an average position of ^1^/_8_ a cell length. In addition, cells lacking MinCD can also position the sporulation septum close to the mid-cell site, with lower frequency and precision. (F) This model shows how the Z- and E-ring can recognize the asymmetric site of septation within a narrow window formed by the negative cell division regulators RefZ and MinCD. However, the possible existence of an additional positive regulator, which helps to form the Z-ring specifically at this site cannot be ruled out.

Despite that RefZ seems to be part of a mechanism important for precisely localizing the sporulation septum, it has not previously been shown where the asymmetric septa localize in cells lacking it. For this reason, we determined the localization of septa in IB1723, a *ΔrefZ*, *spoIIE-Ypet* strain (Materials and Methods). We used the localization of a SpoIIE-Ypet fusion to identify the asymmetric septation sites. We found that the septa appear at 0.20 ± 0.05 of a cell length (mean Ypet position ± SD; 132 cells) from the nearest cell pole, which is ^1^/_5_ of the cell length (Fig 1E). In this deletion strain, 72% of cells positioned the septa within ± 0.05 of the average position of septa as in wild type strain. All these results indicate that cells without RefZ position the asymmetric septation site further away from the poles, but with similar precision. The cells of IB1723 have similar lengths as the wild type cells, with an average cell length of 2.54 ± 0.4 μm compared to 2.46 ± 0.4 μm for the wild type. The average septum location is 0.51 ± 0.17 μm from the pole compared to 0.45 ± 0.12 μm for the wild type (Table 1). The linear correlation coefficient between septum position and cell length is 0.18 (Fig 2C). Taken together, it appears that RefZ has moderate influence on localization of sporulation septum and without its presence the position of septation moves farther from the cell pole, on average from ^1^/_6_ to ^1^/_5_ of the cell length; the cell lengths and correlation coefficients are also similar to the wild type (Fig 3A and 3C).

### Asymmetric septum positioning in the absence of MinCD

Although, it was shown previously that depletion of the MinCD proteins has no detectable effect on sporulation frequency [18], it is still not possible to exclude the possibility that the Min system has a role in positioning the sporulation septum. This is based on the examination of the *minCD* mutant cells where in some cells, a sporulation-like septum appears, but is misplaced from its normal polar site: in these cells it forms either at or near the centre of the cell [19,20]. While the Min system seems to be part of a mechanism, which is important for precisely localizing the sporulation septum, it had not previously been shown where the asymmetric septa localize in cells lacking it. For this reason, we determined the localization of septa in IB1724, a *ΔminCD*, *spoIIE-Ypet* strain (Materials and Methods). We used the localization of a SpoIIE-Ypet fusion to identify sporulation specific septation sites. We identified clear, straight-line localizations of SpoIIE-Ypet in 118 cells. We detected an asymmetrically positioned sporulation septum in 92% of all cells (109 of 118 cells); in 16% of cells (19 of 118) we detected signals at or near the centre of the cell. These two groups were separated for statistical analysis. In the first group we found that the septa appear at 0.115 ± 0.04 of a cell length (mean Ypet position ± SD; 109 cells) from the nearest cell pole, which is ^1^/_8_ of the cell length (Fig 1F). 15% of the cells from this first group had two asymmetrically positioned septa, but all of these were measured from the nearest pole. In this first group, 41% of cells positioned the septa within ± 0.05 of the average septum position as in the wild type strain. These results indicate that cells without MinCD position the asymmetric septa closer to the poles and generally with lower precision. Cells lacking MinCD, which localize the sporulation septa close to the poles, are slightly longer than wild type cells, with an average cell length of 3.05 ± 0.7 μm compared to 2.46 ± 0.4 μm for the wild type. The absolute average septum location is 0.34 ± 0.12 μm from the pole compared to 0.45 ± 0.12 μm for the wild type (Table 1). The linear correlation coefficient between position and cell length is -0.30 which is closer to the *ΔspoIIE* strain than to the wild type strain (Fig 2A, 2B and 2D). Taken together, it appears that the absence of MinCD influences the localization of the sporulation septum, and that in cells without these proteins the septum forms much closer to the cell pole, an average of ^1^/_8_ of a cell length, while the cell is moderately longer than those of the wild type strain (Fig 3A and 3D).

In the second group of *ΔminCD* cells, 16% of the total, we detected signals at or near the centre of the cell. In this group we found that the septa appear at almost ^1^/_2_ (0.48 ± 0.07) of a cell length (mean Ypet position ± SD; 19 cells) (Fig 1F and 1G). In this group, only 42% of cells positioned their septa within ± 0.05 of the mid-cell site. This result indicates that the cells without MinCD can position the sporulation septum near the mid-cell site with low precision. Those cells without MinCD, which localize the sporulation septa close to the mid-cell, are all much longer than wild type cells, with an average cell length of 4.15 ± 1.3 μm compared to 2.46 ± 0.4 μm for the wild type.

We also investigated if EzrA, an additional regulator of vegetative cell division, influences the positioning of the asymmetric septum. Therefore we prepared the strain harboring SpoIIE-Ypet in *ΔezrA* background (IB1725). However, due to the cell division defects of this mutant during vegetative growth, it is difficult to make similar statistics of localization of the sporulation septa and thus unambiguously clarify the role of EzrA in asymmetric cell division (data not shown).

## Discussion

The mechanisms for positioning the division site at mid-cell in *B*. *subtilis* and *E*. *coli*, two model rod shaped bacteria, have been studied for decades (reviewed in [1,32,33]). These studies have shown that the two bacteria find the mid-cell septation sites primarily using the combined action of two negative regulators of Z-ring formation, nucleoid occlusion and the Min system (Fig 3). Interestingly, the newest studies have shown that the Z-ring can be positioned precisely at the center of the *B*. *subtilis* cell even in the complete absence of these two systems [12], leading to the suggestion that some, presently unknown, positive signal or other structure must mark the mid-cell position for Z-ring assembly. It was hypothesized that this factor might be a specific protein that positively regulates Z-ring formation at this site, as recently shown for MapZ in *Streptococcus pneumonia* [34], or it might “potentiate” the mid-cell division site for Z-ring formation as the initiation of DNA replication progresses [35]. There is an even larger gap in our understanding of how the cell finds the specific site of septation for asymmetric division during sporulation in *B*. *subtilis*. The first clear morphological feature of sporulation in *B*. *subtilis* is the polar cell division that starts with migration of the Z-ring from mid-cell to the two cell poles on spiral trajectories [21]. SpoIIE E-rings co-localize with polar Z-rings at these sites (Fig 3). One of the Z-rings matures into the sporulation septum while the other dissolves. Z-ring formation at these sites has to overcome both the negative effects of the Min system, whose proteins also localize at the cell poles, and the nucleoid occlusion system, which would also cover the cell poles since the nucleoids reach both ends of the cell during this early sporulation stage [36]. The switch to polar cell division during sporulation has an interesting consequence for chromosome segregation. At the beginning of sporulation, instead of segregating, the two chromosomes form an elongated structure known as the axial filament [37]. The sporulation septum bisects the axial filament leaving about only one-third of one chromosome in the forespore, and creating a transient genetic asymmetry [38,39]. The remaining two-thirds of the chromosome is then transferred, over a period of 10–20 minutes, from the mother cell into the forespore by a conjugation-like mechanism directed by the SpoIIIE partitioning protein [40]. Spatial localization of the two chromosomes is predetermined by the specific binding of both their *ori* regions to the poles through a DivIVA–RacA protein interaction [16,41].

From studies of division site recognition during the vegetative growth of *E*. *coli* and *B*. *subtilis*, it is clear that rod-shaped bacteria are able to determine the mid-cell site with high precision. However, the site of asymmetric septum formation during *B*. *subtilis* sporulation had not previously been determined. In this study, we concretely identify the actual location of the site of asymmetric septum formation. We show that the asymmetric septum forms around ^1^/_6_ of a cell length from one of the cell poles with high precision (Fig 1). These findings raise at least three crucial questions for bacterial asymmetric cell division. First, why is it important to asymmetrically position the septum during endospore formation in *B*. *subtilis*? Second, why is the asymmetric septum localized at ^1^/_6_ of a cell length from one of the cell poles? Finally, how does the cell recognize this particular site with relatively high precision?

The answer to the first question is based upon the findings that spatial morphological asymmetry is required for a corresponding asymmetry in gene expression in the smaller forespore and larger mother cell during *B*. *subtilis* sporulation [42]. SpoIIE is a crucial protein for asymmetric cell division and for activating the first compartment specific sigma factor, σ^F^. It was hypothesized that the smaller forespore volume leads to a higher specific activity for the SpoIIE phosphatase in the forespore, thereby allowing σ^F^ activation only in this part of the cell [43]. A more plausible reason why an asymmetric septum has to form across one of the chromosomes involves the proposed transient genetic asymmetry at the onset of sporulation. This causes a disproportionate concentration of σ^F^ activation regulators in the mother cell and in the forespore, represented by either a proposed SpoIIE inhibitor [39] or SpoIIAB, both at higher relative levels in the mother cell [44]. All of the players in the mechanisms of σ^F^ activation are conserved in all species of *Bacillus* and *Clostridium* whose genomes have been sequenced to date, suggesting that transient genetic asymmetry is a general mechanism of gene regulation in these bacteria. This would explain why an asymmetric cell division is required for sporulation. On the other hand, the coccoid-shaped *Sporosarcina ureae* divides symmetrically at the onset of sporulation [45], and it remains to be determined how this organism compartmentalizes its gene expression.

The second question, why the sporulation septum is located around ^1^/_6_ of a cell length from one of the cell poles, is more difficult to answer. One possible answer, related to the answer to the first question, is that this precise localization creates an exact volume ratio between the forespore and the mother cell, which could then lead to efficient activation of σ^F^ in a compartment specific manner. Another possibility why the sporulation septum needs to be localized a constant fraction of the cell length the cell pole is that a specific volume and surface is required to form an endospore efficiently, and these parameters are set by the position of the asymmetric septum. A *B*. *subtilis* spore is covered with about 70 different coat proteins, organized in multilayered structures. These proteins have the ability to generate ordered one-dimensional fibres, two-dimensional sheets and three-dimensional stacks, as has been shown by cryo-TEM [46]. At least some of these proteins cover the entire spore surface to form a shield which has astonishing longevity and resistance to environmental insults. For example, CotY was shown to form a double-layered sheet of 6 nm hexameric rings when expressed heterologously in *E*. *coli* [46]. The surface area of an ovoid *B*. *subtilis* spore is around 4–5 μm^2^ and the cell requires a few hundred thousand CotY subunits to continuously cover the spore surface with two layers. The spore coat proteins are some of the most heavily expressed proteins in *B*. *subtilis* cells [47], and the expression of these proteins is likely at the cell’s maximum limit for building the spore coat efficiently.

The final question, how does the cell recognize this particular site with relatively high precision is, at least partially, answered in this study. Our results show that SpoIIE is an important determinant of asymmetric division site positioning: SpoIIE absence not only decreases the efficiency of asymmetric septum formation, but also causes the septation site to shift from ^1^/_6_ of a cell length, to ^1^/_4_ of a cell length, with a concomitant loss of precision (Fig 3). These results indicate that FtsZ without SpoIIE can still promote asymmetric division, but with a lower precision and at a different site. It was also shown previously that the ultrastructure of the septum formed differs in the SpoIIE deletion strain, resembling the thicker vegetative septum [22,30]. RodZ, as a SpoIIE binding partner, has been shown to help stabilize it at the specific asymmetric division site [23]. Taken together, SpoIIE is the only protein known with such a profound role in asymmetric division site formation. Nevertheless, how SpoIIE helps to recognize the cell division site is not understood even remotely. We hypothesize that SpoIIE can coordinate the localization of the protein complexes of the divisome (through interactions with FtsZ and DivIVA), the elongasome (through interaction with RodZ), and probably the localization of the chromosome segregation machinery. Nevertheless, this site might be predetermined by the chromosomal arrangement. It is known that specific nucleoid region is always captured in the forespore, and it has been shown that RefZ, a DNA-binding protein, is important for delineating this region. **R**efZ binds to cognate **b**inding **m**otifs (RBMs) which flank the region of the chromosome captured during cell division [24]. RefZ and the RBMs contribute to determining the relative positioning of the chromosomal arms with respect to the asymmetric division plane. Surprisingly, all published studies suggest that RefZ acts as a negative regulator of Z-ring assembly [24,25], meaning that, while RefZ might influence the absolute positioning of the Z-ring with respect to specific chromosomal regions, it could also inhibit additional Z-ring formation at the same pole. Altogether, the localization of asymmetric septum ^1^/_6_ of a cell length from the cell pole might be predetermined by a specific chromosome arrangement, but the involvement of other mechanisms cannot be excluded. In this study we show that the absence of RefZ in the cell leads to a shift of the asymmetric septation site from an average position of ^1^/_6_ a cell length to ^1^/_5_. If RefZ functions as a negative regulator of Z-ring assembly, then this finding suggests that RefZ blocks septation sites further than ^1^/_6_ of a cell length from the cell pole, but it does not allow us to exclude the possibility that RefZ also blocks septation closer to the poles. The Min system has an even more profound effect on asymmetric septum site positioning. We determined that the absence of MinCD in the cells causes the average position of the septation site to move much closer to the cell pole: to an average of ^1^/_8_ of a cell length in from the wild type value of ^1^/_6_ a cell length. Together, these results suggest that the Min system efficiently blocks sporulation septum formation close to the poles while RefZ blocks formation further away than ^1^/_6_ of a cell length at this particular stage of development. The cell division machinery, together with the crucial SpoIIE protein, seems to recognize the narrow available space between these two negative cell division regulators (Min system and RefZ), and it is only at this site that the sporulation septum can be formed with high precision (Fig 3F). Unfortunately, we cannot rule out the possible existence of an additional positive regulator, which might promote Z-ring formation specifically at this site.

How is the asymmetric site of septation determined in other bacteria? It is likely that in other rod-shaped endospore forming bacteria, in other Bacilli and Clostridia species, the mechanisms of site recognition are similar since they have homologues of most of the important *B*. *subtilis* proteins, including SpoIIE, RodZ, DivIVA, Min system and RefZ. All the available data show that this is a very complex mechanism with many different proteins and protein complexes, including the divisome, elangasome and specific chromosome segregation machinery.
